# The first patient-reported outcomes from the Utrecht Prostate Cohort (UPC): the first platform facilitating ‘trials within cohorts’ (TwiCs) for the evaluation of interventions for prostate cancer

**DOI:** 10.1007/s00345-022-04092-2

**Published:** 2022-07-21

**Authors:** Frederik R. Teunissen, Thomas Willigenburg, Richard P. Meijer, Harm H. E. van Melick, Helena M. Verkooijen, Jochem R. N. van der Voort van Zyp

**Affiliations:** 1 Department of Radiation Oncology, University Medical Center Utrecht, Heidelberglaan 100, 3584 CZ Utrecht, The Netherlands; 2 Department of Oncologic Urology, University Medical Center Utrecht, Utrecht, The Netherlands; 3 Department of Urology, St. Antonius Hospital, Nieuwegein, Utrecht, The Netherlands; 4 Imaging Division, University Medical Center Utrecht, Utrecht, The Netherlands; 5 Utrecht University, Utrecht, The Netherlands

**Keywords:** Prostatic neoplasms, Cohort studies, Patient-reported outcome measures, Treatment outcome, Quality of life

## Abstract

**Purpose:**

To describe the development and first outcomes of the Utrecht Prostate Cohort (UPC): the first ‘trials within cohorts’ (TwiCs) platform for prostate cancer (PCa).

**Methods:**

All non-metastasized, histologically proven PCa patients who are planned to receive standard of care are eligible for inclusion in UPC. Patients provide informed consent for the collection of clinical and technical patient data, physician-reported outcomes, and patient-reported outcomes (PROs) up to 10 years post-treatment. Additionally, patients may provide broad consent for future randomization for experimental-intervention trials (TwiCs). Changes in PROs (EPIC-26 questionnaire domains) of the participants who received standard of care were analyzed using Wilcoxon signed-rank tests.

**Results:**

In two years, 626 patients were enrolled, 503 (80.4%) of whom provided broad consent for future randomization. Among these, 293 (46.8%) patients underwent magnetic resonance-guided adaptive radiotherapy (MRgRT), 116 (18.5%) CT-guided external beam radiation therapy (EBRT), 109 (17.4%) robot-assisted radical prostatectomy (RARP), and 65 (10.4%) patients opted for active surveillance. Patients treated with MRgRT and CT-guided EBRT showed a transient but significant decline in urinary irritative/obstructive and bowel domain scores at 1-month follow-up. RARP patients showed a significant deterioration of urinary incontinence domain scores between baseline and all follow-up moments and significant improvement of urinary irritative/obstructive domain scores between baseline and 9- and 12-month follow-up. All radical treatment groups showed a significant decline in sexual domain scores during follow-up. Active surveillance patients showed no significant deterioration over time in all domains.

**Conclusion:**

The first results from the UPC study show distinct differences in PROs between treatment options for PCa.

Registration No.: NCT04228211.

**Supplementary Information:**

The online version contains supplementary material available at 10.1007/s00345-022-04092-2.

## Introduction

Prostate cancer (PCa) is the most common cancer in men worldwide, with an estimated 1,414,259 new cases and 375,304 associated deaths in 2020 [1]. Overall survival rates are high due to the non-aggressive nature of many localized prostate tumors and the availability of effective treatment options [2].

Established curative (radical) treatment modalities for primary localized PCa include external beam radiation therapy (EBRT), brachytherapy (BT), and robot-assisted radical prostatectomy (RARP). Radical treatments are associated with adverse events such as genitourinary (GU) and gastrointestinal (GI) problems, and erectile dysfunction (ED). Therefore, new treatment modalities, aimed at reducing adverse events and improving quality of life (QoL), are being developed. These include real-time magnetic resonance-guided radiotherapy (MRgRT) and focal therapies such as high-intensity focused ultrasound (HIFU) or irreversible electroporation (IRE) [3–5]. An alternative to active treatment for selected low- and intermediate-risk PCa patients is active surveillance (AS).

Evaluation of new treatments is ideally performed using the randomized controlled trial (RCT) design. RCTs are often limited by slow recruitment, high rates of premature ending [6], limited generalizability due to strict patient inclusion criteria that may not represent the real-world patient population [7, 8], fear for the experimental treatment, which can prevent patients from participating, or preference for the new intervention, leading to patient disappointment and even drop out as a result of allocation to the control arm [9]. To overcome some of these limitations, the trials within cohorts (TwiCs) design was developed [10]. In this TwiCs design, prospective cohorts or registries serve as facilities for simultaneous and randomized evaluation of multiple interventions for the same condition. The basis of TwiCs is a comprehensive prospective observational cohort of patients with the condition of interest (e.g., PCa), who (in principle) undergo standard treatment and for whom relevant short- and long-term outcome measures are captured. For each experimental intervention that is compared to standard treatment in an RCT, a subcohort of eligible patients is identified within the cohort. From this subcohort of eligible patients, a random sample is offered the intervention. The outcomes of these randomly selected patients are then compared to the remaining eligible patients in the subcohort who received standard care. During the trial, the control group is not actively informed about the trial. The same process can be repeated (simultaneously) for other experimental interventions [11, 12].

Due to the high pace of technical innovations in PCa treatment, we set up a comprehensive cohort of patients with non-metastasized, histologically proven PCa, facilitating the TwiCs design: the “Utrecht Prostate Cohort for cancer treatment intervention studies and long-term evaluation” (UPC). With UPC, we aim to: (1) create a real-life data infrastructure for the evaluation of short- and long-term clinical and patient-reported outcomes during and after treatment for PCa. (2) Provide a facility for multiple interventional trials and observational studies for the evaluation of new treatment interventions for PCa. This paper describes the infrastructural set up and presents the first data from all patients enrolled in the UPC study in the first two years of inclusion.

## Materials and methods

### Patients

The UPC study received approval from the Institutional Review and Ethics Board of the University Medical Center Utrecht (19-692/M), the Netherlands. All non-metastatic, histologically proven PCa patients are eligible for participation in UPC. After diagnosis, patients are informed by a researcher or research assistant about the study, after which written informed consent is obtained. Patients that are mentally incompetent or unable to understand the Dutch language are excluded from participation. Enrolment takes place at two urology clinics and one radiotherapy facility covering a large region within the Netherlands.

### Staged-informed consent

In addition to signing informed consent for the collection, use, and sharing of clinical and technical data and receiving QoL questionnaires, patients may provide broad consent for random allocation to experimental interventional treatment(s) in the (near) future in case they are eligible for a trial within the cohort [10, 11]. In this case, patients who are randomly allocated to the experimental arm are offered to undergo an experimental treatment, for which, in case they accept, additional written informed consent is obtained. Patients allocated to the control arm will receive standard treatment and are not informed while the study is ongoing. According to the TwiCs design, multiple trials can run within UPC simultaneously. All patients are informed about the results after completion of a study within UPC, irrespective of their participation in that specific study.

### Clinical data

For the observational cohort, clinical data are prospectively collected and stored in a cloud-based database. Data are collected from the electronic patient records, referral letters, and annual data extraction from the Dutch cancer registry.

Sociodemographic data include: date of birth, family history of PCa, educational level, Charlson Comorbidity Index (CCI), and Eastern Cooperative Oncology Group (ECOG) performance status. Disease characteristics include: date of diagnosis, PSA level, tumor nodes metastases (TNM) classification, pathological results, prostate volume, prostate imaging reporting and data system (PI-RADS) classification, prostate-specific membrane antigen ligand positron emission tomography (PSMA-PET) computed tomography (CT) results, and bone scintigraphy. Imaging data are stored in a Digital Imaging and Communications in Medicine (DICOM) repository. For patients undergoing RARP and/or pelvic lymph node dissection, additional pathologic information is collected, including pathologic tumor and lymph node status and surgical margins. Surgical complications are recorded using the Clavien Dindo classification. For radiotherapy patients, irradiated volume, prescribed dose, and documentation of androgen deprivation therapy prescription are collected. Acute and chronic toxicity is collected using the National Cancer Institute’s Common Toxicity Criteria for Adverse Events (CTCAE) version 5.

Recurrence- and progression-free survival are assessed following routine care by regular measurement of PSA level. Survival is assessed through follow-up questionnaires, the systematical assessment of the Municipal Personal Records Database, and the Dutch Cancer Registry.

### Patient-reported outcomes (PROs)

Patients have the option to fill out paper QoL questionnaires or opt for online completion of the QoL questionnaires after secured login. Patients are invited to fill out questionnaires at baseline and at 1, 3, 6, 9, 12, 18, and 24 months post-treatment. Thereafter, questionnaires are filled out annually up to 10 years post-treatment. Annually, additional information is obtained on (serious) adverse events.

PRO questionnaires include: Expanded Prostate Cancer Index Composite Short Form (EPIC-26) [13], EORTC Quality of Life Questionnaire (QLQ-C30) [14], International Index of Erectile Function-5 (IIEF-5) [15], EuroQol questionnaire (EQ-5D-5L) [16], International Prostate Symptom Score (IPSS) [17], Hospital Anxiety and Depression Scale (HADS) [18], and Workability Index (WAI) [19] (the WAI questionnaire is not included at 1, 6, 9 and 18 months).

### Statistical analysis

Descriptive statistics were reported for the questionnaire response rate, baseline characteristics, and the outcomes of the EPIC-26 questionnaire at baseline, 1, 3, 6, 9, and 12 months follow-up for each major treatment group. For the AS patient group, the first completed questionnaire was set as baseline questionnaire. Within each treatment group, follow-up EPIC-26 scores were compared to baseline using the difference in medians (Δ) and Wilcoxon signed-rank tests. A *p*-value < 0.05 was considered statistically significant. The minimal clinically relevant difference for the EPIC-26 domain scores was considered Δ = 5–7 for the urinary irritative/obstructive domain, Δ = 6–9 for the urinary incontinence domain, Δ = 4–6 for the bowel domain, Δ = 10–12 for the sexual domain, and Δ = 4–6 for the hormonal domain [20]. All analyses were performed using R version 4.1.2.

## Results

Between February 5th, 2020, and February 5th, 2022, 626 patients were enrolled in UPC. All participants signed informed consent for the use of their data for research purposes, 556 (88.8%) provided consent for filling out PRO questionnaires, and 503 (80.4%) provided broad consent for future randomization (TwiCs). Since the start of the study, two (0.3%) patients withdrew from participation, and nine (1.4%) patients deceased during follow-up.

On February 5th, 2022, 293 (46.8%) patients had started or completed MRgRT treatment, 116 (18.5%) had started or completed CT-guided EBRT treatment, and 109 (17.4%) patients underwent RARP. An additional 65 (10.4%) patients opted for AS (Table 1). Patients who underwent RARP were youngest on average (mean: 68.2 years), followed by those who opted for AS (mean: 68.4 years), MRgRT (mean 70.3 years), and CT-guided EBRT (mean 73.0 years). Most RARP and MRgRT patients had intermediate-risk localized PCa (53.2 and 74.1%, respectively), whereas most AS patients had low-risk localized PCa (70.8%). In the CT-guided EBRT group, most patients had high-risk localized PCa (72.4%).

**Table 1:** Baseline characteristics of the four major patient groups within the UPC study

Characteristic	Treatment group			
	MRgRT	CT-guided EBRT	RARP	AS
*n*	293	116	109	65
Age (Mean (SD))	70.3 (6.4)	73.0 (6.2)	68.2 (5.7)	68.4 (6.1)
Charlson comorbidity index (Mean (SD))	0.6 (1.1)	0.8 (1.2)	0.3 (0.8)	0.4 (0.7)
Consent for receiving PRO questionnaires (*n* (%))	264 (90.1)	90 (77.6)	104 (95.4)	61 (93.8)
Fraction scheme (*n* (%))				
5 × 7.25 Gy	243 (82.9)	8 ( 6.9)	NA	NA
20 × 3.1 Gy	47 (16.0)	61 (52.6)	NA	NA
35 × 2.2 Gy	0 (0.0)	40 (34.5)	NA	NA
Other	3 (1.0)	7 (6.0)	NA	NA
ADT (*n* (%))	40 (13.7)	83 (72.8)	3 (2.8)	0 (0.0)
cT stage (*n* (%))				
cT1	150 (51.4)	36 (31.0)	57 (52.3)	53 (81.5)
cT2	128 (43.8)	39 (33.6)	39 (35.8)	11 (16.9)
cT3	13 (4.5)	40 (34.5)	13 (11.9)	1 (1.5)
cT4	1 (0.3)	1 (0.9)	0 (0.0)	0 (0.0)
Regional lymph node metastasis (*n* (%))	2 (0.1)	28 (24.1)	7 (6.4)	0 (0.0)
PSA (*n* (%))				
< 10 ng/ml	29 (9.9)	5 (4.3)	11 (10.1)	46 (70.8)
10–20 ng/ml	217 (74.1)	27 (23.3)	58 (53.2)	17 (26.2)
> 20 ng/ml	47 (16.0)	84 (72.4)	40 (36.7)	2 (3.1)
Gleason score (*n* (%))				
≤ 6	48 (16.4)	8 ( 6.9)	19 (17.4)	54 (83.1)
7	225 (76.8)	49 (42.3)	65 (59.7)	11 (16.9)
≥ 8	20 ( 6.8)	59 (50.9)	25 (23.0)	0 (0.0)
Risk classification (EAU) (*n* (%))				
Low risk	29 (9.9)	5 (4.3)	11 (10.1)	46 (70.8)
Intermediate risk	217 (74.1)	27 (23.3)	58 (53.2)	17 (26.2)
High risk	47 (16.0)	84 (72.4)	40 (36.7)	2 (3.1)

*UPC *Utrecht prostate cohort, *MRgRT* magnetic resonance-guided adaptive radiotherapy, *EBRT* external beam radiotherapy, *RARP* robot assisted radical prostatectomy, *AS* active surveillance, *PRO* patient-reported outcome, *ADT* androgen deprivation therapy, *cT stage* clinical tumor stage, *PSA* prostate-specific antigen, *EAU* European association of urology

**Fig. 1:**
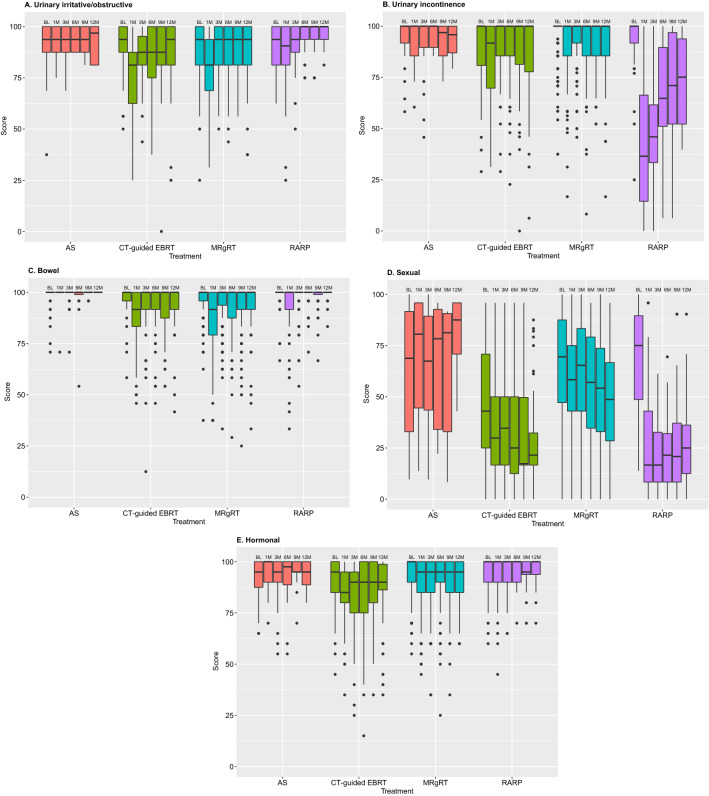
EPIC-26 domain scores for the four largest patient groups at baseline and 1, 3, 6, 9, and 12 months follow-up (numbers at risk in supplementary material). *BL* baseline, *M* month, *AS* active surveillance, *EBRT *external beam radiation therapy, *MRgRT* magnetic resonance-guided adaptive radiotherapy, *RARP* robot-assisted radical prostatectomy

The questionnaire response rate for the entire cohort was 78.8% at baseline, 76.7% at 6-month follow-up, and 71.0% at 12-month follow-up. For the EPIC-26 urinary irritative/obstructive domain, the MRgRT and CT-guided EBRT patients reported significant and clinically relevant lower scores at 1-month follow-up compared to baseline (MRgRT: Δ-12.5, *p* < 0.001; CT-guided EBRT: Δ–12.5, *p* < 0.001) (Fig. 1 and supplementary material). RARP patients reported a significant and clinically relevant improvement in urinary irritative/obstructive domain scores at 9- (Δ + 6.2, *p* = 0.002) and 12-month (Δ + 6.2, *p* = 0.029) follow-up compared to baseline. In these patients, a significant and clinically relevant decline in the urinary incontinence domain scores at 1, 3, 6, 9, and 12-month follow-up compared to baseline was reported (Δ–63.5, *p* < 0.001; Δ–54.0, *p* < 0.001; Δ–35.3, *p* < 0.001; Δ–29.0, *p* = 0.001; Δ–24.9, *p* = 0.045; respectively) and at 1-month follow-up by the CT-guided EBRT patients (Δ–8.2, *p* = 0.031). For the bowel domain, the MRgRT and CT-guided EBRT patients reported significant and clinically relevant lower scores at 1-month follow-up compared to baseline (MRgRT: Δ–8.3, *p* < 0.001; CT-guided EBRT: Δ–8.3, *p* < 0.001). The median sexual domain score declined significantly from baseline up to 12-month follow-up from 43.0 to 21.5 (*p* < 0.001) for the CT-guided EBRT group, from 69.5 to 48.7 (*p* < 0.001) for the MRgRT group, and from 75.0 to 25.0 (*p* < 0.001) for the RARP group. In the MRgRT and CT-guided EBRT group, a significant and clinically relevant decline of the hormonal domain score from baseline was observed for all follow-up moments and for the RALP patients at 9-month follow-up. In the AS group, no significant difference in any of the domains at any follow-up point was observed.

## Discussion

New treatments for PCa patients are being developed at a rapid pace. Multiple (simultaneous) trials or other studies for new treatment interventions can be conducted within UPC. We prospectively collect a predefined set of baseline and follow-up measurements for the cohort at regular time points. Using standardized PROs, we can effectively compare short- and long-term outcomes of treatment interventions to standard care, which will be important for the implementation of new treatment interventions in clinical practice. All future trials within UPC will use the same predefined study population, and baseline and follow-up data will be collected at the same time points. This will enable direct comparison between standard-of-care and new treatment interventions and is in line with the International Consortium for Health Outcome Measures (ICHOM), which focuses on the standardization of outcome measures [21]. Next to TwiCs, the UPC study facilitates non-randomized comparison studies between the patient groups within the cohort, as well as with external cohorts. Because of the vast and detailed patient characteristics, treatment procedures, and outcomes collected within UPC, the data can be used for post-marketing studies, technical development studies (following the R-IDEAL framework [22]), prediction studies, and imaging studies.

Across the different standard treatment groups within UPC, different patterns in EPIC-26 domain scores are manifest. Initial analysis of the EPIC-26 domain scores showed no significant difference for any follow-up time points compared to baseline for all domains in the AS group. All radical treatment options showed significant and clinically relevant change in one or more domains at one or more follow-up moments, which is in line with large prospective cohorts in literature [23–26] and affirm the domains in which improvements can be made in terms of toxicity reduction. A limitation of this first UPC report is the relatively low number of included patients, especially towards longer follow-up, which lowers power for comparisons. Ongoing data collection and follow-up will increase these numbers for each treatment group and will enable us to conduct stratified or matched comparisons between groups within the cohort and with external cohorts, allowing the evaluation of differences in toxicity and efficacy between primary PCa treatments.

The urge to prevent ED after radical treatment has led to the first trial that is currently running within UPC. This single-arm phase II trial investigates the effect of neurovascular sparing MRgRT on erectile function in a localized PCa population (NCT04861194) [27, 28]. Because all study parameters for this trial are already prospectively recorded within UPC (e.g., the IIEF-5 questionnaire for the measurement of erectile function), it can run very efficiently. Furthermore, UPC data are currently being used to analyze dose-toxicity relationships in MRgRT patients to evaluate and possibly adjust existing or propose new dose constraints to further reduce toxicity after radiotherapy. Systematically recorded physician- and patient-reported toxicity (i.e., CTCAEs and PROs) and technical data (i.e., MRgRT dose parameters) recorded within UPC are used for this goal.

Currently, only non-metastatic PCa patients that opt for AS or awaiting radical treatment are included. This can be extended to additionally include PCa patients with metastatic or recurrent disease undergoing palliative or salvage treatments. Also, patients at risk of PCa can be included to analyze diagnostic strategies before the diagnosis of PCa. Furthermore, a biobank for genetic and (histo) pathologic studies will be added in the near future. The UPC study is designed in such a way that it can be expanded to other medical centers, and external institutions can apply to receive data for research purposes.

The TwiCs design overcomes some of the hurdles that are associated with running classic RCTs. Advantages of the TwiCs design over the classic RTC design include more efficient use of control patients, improved comparison between different trial interventions, enhanced generalizability, and reduced disappointment bias [10, 12]. However, there are some limitations of TwiCs design. First, the collected clinical data are generated from routine care and, therefore, may be considered pragmatic. Endpoints for trials within UPC need to be part of the predefined outcomes measured for all patients. Second, the questionnaire return rates slowly decrease over time, which is also a concern in the UPC study and may influence data comparability and generalizability. Therefore, we are actively informing patients about the results of studies conducted within the cohort to keep participants actively involved and motivated to return the questionnaires. Third, in the TwiCs design, a patient allocated to the control arm is not informed about being a participant in a trial and is also not informed about the interventional treatment. The (conventional) control treatment can be considered the best treatment in terms of outcome based on the current, up-to-date guidelines. However, because a patient is withheld the information and possibly unaware of the existence of a specific experimental treatment, the patient does not have the opportunity to receive the experimental treatment off-protocol or outside a clinical trial. Although UPC participants sign informed consent for these procedures up-front, therapeutic misconception could remain an issue since optimism about potentially being randomized in the experimental arm when participating in UPC could overshadow the equal chance of being randomized in the control arm and the even higher chance of not participating in a trial at all [11, 29]. Therefore, researchers should extensively inform participants about this TwiCs procedure before participants sign consent [30].

## Conclusion

The UPC study is the first platform for PCa according to the TwiCs design. It provides an ongoing prospective observational cohort and an infrastructure for multiple trials and other studies for the evaluation of new treatment interventions for PCa. The initial results after two years of inclusion highlight the areas on which future research and new interventions should focus.

## Supplementary Information

Below is the link to the electronic supplementary material.

Supplementary file1 (DOCX 33 KB)
