# Supplementary material for: The first patient-reported outcomes from the Utrecht Prostate Cohort (UPC): the first platform facilitating ‘trials within cohorts’ (TwiCs) for the evaluation of interventions for prostate cancer
Source: World J Urol. 2022 Jul 21;40(9):2205–12. doi: 10.1007/s00345-022-04092-2 (PMC9427931; doi:10.1007/s00345-022-04092-2)
Supplement: Supplementary file 1 — Supplementary file1 (DOCX 33 KB) [file 345_2022_4092_MOESM1_ESM.docx]

**Supplementary material**

EPIC-26 outcomes

A. Urinary irritative/obstructive

|  |  | n | Median | IQR | p-value* | Effect size* |
| --- | --- | --- | --- | --- | --- | --- |
| CT-guided EBRT | BL | 64 | 93.8 | 87.5 – 100 |  |  |
|  | 1M | 75 | **81.3** | 62.5 - 87.5 | **< 0.001** | **0.61** |
|  | 3M | 72 | **87.5** | 81.3 – 95.3 | 0.255 | 0.15 |
|  | 6M | 69 | **87.5** | 75.0 – 100 | 0.659 | 0.03 |
|  | 9M | 56 | **87.5** | 81.3 – 100 | 0.051 | 0.28 |
|  | 12M | 46 | 93.8 | 81.3 – 100 | 0.488 | 0.10 |
| AS | BL | 47 | 93.8 | 87.5 - 100 |  |  |
|  | 1M | 33 | 93.8 | 87.5 - 100 | 0.462 | 0.20 |
|  | 3M | 27 | 93.8 | 87.5 - 100 | 0.809 | 0.02 |
|  | 6M | 16 | 93.8 | 87.5 - 100 | 0.145 | 0.35 |
|  | 9M | 14 | 93.8 | 87.5 - 100 | 0.572 | 0.14 |
|  | 12M | 8 | 96.9 | 81.3 - 100 | 0.233 | 0.47 |
| MRgRT | BL | 183 | 93.8 | 81.3 - 100 |  |  |
|  | 1M | 214 | **81.3** | 68.8 - 93.8 | **< 0.001** | **0.60** |
|  | 3M | 171 | 93.8 | 81.3 - 100 | 0.745 | 0.02 |
|  | 6M | 149 | 93.8 | 81.3 - 100 | 0.240 | 0.09 |
|  | 9M | 119 | 93.8 | 81.3 - 100 | 0.741 | 0.02 |
|  | 12M | 83 | 93.8 | 81.3 - 100 | 0.340 | 0.15 |
| RARP | BL | 67 | 93.8 | 81.3 - 100 |  |  |
|  | 1M | 68 | 90.6 | 81.3 - 100 | 0.026 | 0.30 |
|  | 3M | 55 | 93.8 | 87.5 - 100 | 0.784 | 0.07 |
|  | 6M | 47 | **100** | 93.8 - 100 | 0.286 | 0.17 |
|  | 9M | 44 | **100** | 93.8 - 100 | **0.002** | **0.51** |
|  | 12M | 24 | **100** | 93.8 - 100 | **0.029** | **0.54** |

*for comparison with BL

B. Urinary incontinence

|  |  | n | Median | IQR | p-value* | Effect size* |
| --- | --- | --- | --- | --- | --- | --- |
| CT-guided EBRT | BL | 72 | 100 | 80.8 - 100 |  |  |
|  | 1M | 62 | **91.8** | 69.8 - 100 | **0.031** | **0.32** |
|  | 3M | 75 | 100 | 85.5 - 100 | 0.323 | 0.11 |
|  | 6M | 69 | 100 | 85.5 - 100 | 0.594 | 0.09 |
|  | 9M | 55 | 100 | 81.4 - 100 | **0.002** | **0.47** |
|  | 12M | 46 | 100 | 77.8 - 100 | 0.072 | 0.30 |
| AS | BL | 47 | 100 | 91.8 - 100 |  |  |
|  | 1M | 33 | 100 | 85.5 - 100 | 0.658 | 0.03 |
|  | 3M | 27 | 100 | 89.6 - 100 | 0.350 | 0.10 |
|  | 6M | 15 | 100 | 89.6 - 100 | 0.371 | 0.37 |
|  | 9M | 14 | 96.9 | 85.5 - 100 | 0.731 | 0.02 |
|  | 12M | 8 | 95.9 | 87.0 - 100 | 0.999 | 0.03 |
| MRgRT | BL | 181 | 100 | 100 - 100 |  |  |
|  | 1M | 214 | 100 | 85.5 - 100 | **0.002** | **0.29** |
|  | 3M | 170 | 100 | 91.8 - 100 | 0.340 | 0.04 |
|  | 6M | 149 | 100 | 85.5 - 100 | 0.053 | 0.24 |
|  | 9M | 117 | 100 | 85.5 - 100 | **0.041** | **0.22** |
|  | 12M | 83 | 100 | 85.5 - 100 | **0.048** | **0.28** |
| RARP | BL | 67 | 100 | 91.8 - 100 |  |  |
|  | 1M | 68 | **36.5** | 14.5 – 66.31 | **< 0.001** | **0.85** |
|  | 3M | 55 | **46.0** | 33.4 – 61.62 | **< 0.001** | **0.87** |
|  | 6M | 47 | **64.8** | 51.2 – 89.62 | **< 0.001** | **0.69** |
|  | 9M | 43 | **71.0** | 52.3 - 96.88 | **0.001** | **0.56** |
|  | 12M | 24 | **75.2** | 52.3 – 93.81 | **0.045** | **0.54** |

*for comparison with BL

C. Bowel

|  |  | n | Median | IQR | p-value* | Effect size* |
| --- | --- | --- | --- | --- | --- | --- |
| CT-guided EBRT | BL | 64 | 100 | 95.8 - 100 |  |  |
|  | 1M | 75 | **91.7** | 83.3 - 100 | **< 0.001** | **0.70** |
|  | 3M | 72 | 100 | 91.7 - 100 | **0.006** | **0.35** |
|  | 6M | 69 | 100 | 91.7 - 100 | **0.007** | **0.40** |
|  | 9M | 55 | 100 | 87.5 - 100 | **< 0.001** | **0.58** |
|  | 12M | 46 | 100 | 91.7 - 100 | **0.035** | **0.39** |
| AS | BL | 47 | 100 | 100 - 100 |  |  |
|  | 1M | 33 | 100 | 100 - 100 | 0.856 | 0.17 |
|  | 3M | 27 | 100 | 100 - 100 | 0.892 | 0.08 |
|  | 6M | 16 | 100 | 99.0 - 100 | 0.581 | 0.24 |
|  | 9M | 14 | 100 | 100 - 100 | 0.773 | 0.15 |
|  | 12M | 8 | 100 | 100 - 100 | 0.371 | 0.50 |
| MRgRT | BL | 183 | 100 | 95.8 - 100 |  |  |
|  | 1M | 214 | **91.7** | 79.2 - 100 | **< 0.001** | **0.64** |
|  | 3M | 171 | 100 | 93.8 - 100 | **0.027** | **0.21** |
|  | 6M | 149 | 100 | 87.5 - 100 | **0.002** | **0.31** |
|  | 9M | 119 | 100 | 91.7 - 100 | **0.035** | **0.15** |
|  | 12M | 83 | 100 | 91.7 - 100 | 0.116 | 0.20 |
| RARP | BL | 67 | 100 | 100 - 100 |  |  |
|  | 1M | 68 | 100 | 91.7 - 100 | **< 0.001** | **0.54** |
|  | 3M | 55 | 100 | 100 - 100 | 0.306 | 0.23 |
|  | 6M | 47 | 100 | 100 - 100 | 0.621 | 0.12 |
|  | 9M | 44 | 100 | 99.0 - 100 | 0.796 | 0.06 |
|  | 12M | 24 | 100 | 100 - 100 | 0.999 | 0.12 |

*for comparison with BL

D. Sexual

|  |  | n | Median | IQR | p-value* | Effect size* |
| --- | --- | --- | --- | --- | --- | --- |
| CT-guided EBRT | BL | 61 | 43.0 | 25.0 – 70.8 |  |  |
|  | 1M | 70 | **29.8** | 16.7 – 50.0 | **< 0.001** | **0.69** |
|  | 3M | 65 | 34.7 | 16.7 – 50.0 | **< 0.001** | **0.67** |
|  | 6M | 65 | **25.0** | 12.5 – 50.0 | **< 0.001** | **0.73** |
|  | 9M | 50 | **17.3** | 16.7 – 49.7 | **< 0.001** | **0.75** |
|  | 12M | 44 | **21.5** | 16.7 – 32.3 | **< 0.001** | **0.87** |
| AS | BL | 40 | 68.8 | 33.0 - 91.7 |  |  |
|  | 1M | 26 | 80.6 | 44.5 - 95.8 | 0.936 | 0.06 |
|  | 3M | 22 | 67.4 | 43.5 – 89.4 | 0.586 | 0.16 |
|  | 6M | 12 | 78.3 | 34.1 – 92.7 | 0.675 | 0.21 |
|  | 9M | 10 | 81.4 | 33.0 – 90.6 | 0.234 | 0.42 |
|  | 12M | 5 | 87.5 | 70.8 – 95.8 | 0.999 | 0.06 |
| MRgRT | BL | 174 | 69.5 | 47.2 - 87.5 |  |  |
|  | 1M | 205 | **58.3** | 43.0 – 75.0 | **< 0.001** | **0.51** |
|  | 3M | 169 | 65.3 | 43.0 - 83.3 | **< 0.001** | **0.38** |
|  | 6M | 142 | **57.0** | 34.7 - 79.2 | **< 0.001** | **0.51** |
|  | 9M | 116 | **54.2** | 33.0 – 73.7 | **< 0.001** | **0.58** |
|  | 12M | 78 | **48.7** | 28.5 - 66.7 | **< 0.001** | **0.77** |
| RARP | BL | 55 | 75.0 | 48.7 – 89.6 |  |  |
|  | 1M | 36 | **16.7** | 8.3 – 43.0 | **< 0.001** | **0.74** |
|  | 3M | 47 | **16.7** | 8.3 – 32.7 | **< 0.001** | **0.87** |
|  | 6M | 38 | **21.5** | 8.3 – 32.0 | **< 0.001** | **0.87** |
|  | 9M | 40 | **20.8** | 8.3 – 37.2 | **< 0.001** | **0.87** |
|  | 12M | 21 | **25.0** | 12.5 – 36.2 | **< 0.001** | **0.88** |

*for comparison with BL

E. Hormonal

|  |  | n | Median | IQR | p-value* | Effect size* |
| --- | --- | --- | --- | --- | --- | --- |
| CT-guided EBRT | BL | 64 | 95.0 | 85.0 - 100 |  |  |
|  | 1M | 75 | **85.0** | 80.0 – 95.0 | **< 0.001** | **0.63** |
|  | 3M | 72 | **90.0** | 75.0 – 95.0 | **0.003** | **0.46** |
|  | 6M | 69 | **90.0** | 75.0 – 100 | **< 0.001** | **0.49** |
|  | 9M | 56 | **90.0** | 80.0 – 100 | **0.016** | **0.39** |
|  | 12M | 46 | **90.0** | 86.3 – 98.8 | **0.031** | **0.36** |
| AS | BL | 47 | 95.0 | 87.5 – 100 |  |  |
|  | 1M | 33 | 100 | 90.0 - 100 | 0.979 | < 0.01 |
|  | 3M | 27 | 95.0 | 90.0 - 100 | 0.774 | 0.06 |
|  | 6M | 16 | 97.5 | 88.8 - 100 | 0.323 | 0.22 |
|  | 9M | 14 | 95.0 | 95.0 - 100 | 0.569 | 0.08 |
|  | 12M | 8 | 95.0 | 88.8 - 100 | 0.890 | 0.13 |
| MRgRT | BL | 183 | 100 | 90.0 - 100 |  |  |
|  | 1M | 214 | **95.0** | 85.0 - 100 | **< 0.001** | **0.33** |
|  | 3M | 171 | **95.0** | 85.0 - 100 | **< 0.001** | **0.36** |
|  | 6M | 149 | **95.0** | 90.0 - 100 | **0.013** | **0.23** |
|  | 9M | 119 | **95.0** | 85.0 - 100 | **0.004** | **0.34** |
|  | 12M | 83 | **95.0** | 85.0 - 100 | **0.003** | **0.43** |
| RARP | BL | 67 | 100 | 90.0 - 100 |  |  |
|  | 1M | 68 | 100 | 90.0 - 100 | 0.999 | 0.04 |
|  | 3M | 55 | 100 | 90.0 - 100 | 0.172 | 0.19 |
|  | 6M | 47 | 100 | 90.0 - 100 | 0.594 | 0.15 |
|  | 9M | 44 | **95.0** | 93.8 - 100 | **0.029** | **0.42** |
|  | 12M | 24 | 100 | 93.8 - 100 | 0.394 | 0.20 |

*for comparison with BL

Effect size: < 0.30 = small; 0.30 – 0.49 = moderate; ≥ 0.50 = large [1].

[1] Fritz CO, Morris PE, Richler JJ. Effect size estimates: Current use, calculations, and interpretation. J Exp Psychol Gen 2012. https://doi.org/10.1037/a0024338.
